# High-Quality Draft Genome Sequence of *Pseudomonas* sp. BRG100, a Strain with Bioherbicidal Properties against *Setaria viridis* (Green Foxtail) and Other Pests of Agricultural Significance

**DOI:** 10.1128/genomeA.00995-14

**Published:** 2014-10-02

**Authors:** Tim J. Dumonceaux, Jennifer Town, Matthew G. Links, Sue Boyetchko

**Affiliations:** a Agriculture and Agri-Food Canada, Saskatoon Research Centre, Saskatoon, Saskatchewan, Canada; b Department of Veterinary Microbiology, University of Saskatchewan, Saskatoon, Saskatchewan, Canada; c Department of Computer Science, University of Saskatchewan, Saskatoon, Saskatchewan, Canada

## Abstract

*Pseudomonas* sp. BRG100 inhibits the growth of certain agricultural pests and is a potentially useful biopesticide for weeds and plant diseases. We have sequenced the 6.25-Mbp genome of this strain and assembled it into 4 scaffolds. Genome sequence comparisons revealed that this strain may represent a novel species of *Pseudomonas*.

## GENOME ANNOUNCEMENT

*Pseudomonas* sp. BRG100 (previously reported as *P. fluorescens* BRG100) is a bacterium isolated from the seed and roots of green foxtail (*Setaria viridis*). This organism inhibits the germination and root growth of green foxtail along with fungal plant pathogens such as *Phoma lingam, Rhizoctonia solani, Alternaria brassicae*, and *Sclerotinia sclerotiorum* (1). The herbicidal and fungicidal properties of *Pseudomonas* sp. BRG100 have been attributed to the production of extracellular secondary metabolites such as pseudophomins A and B, which are cyclic lipodepsipeptides (1–3). *In situ* colonization of the roots of *S. viridis* and subsequent growth suppression have been demonstrated using green fluorescent protein-tagged transformants of *Pseudomonas* sp. BRG100, suggesting that this strain may be useful as a biopesticide to control annual grass weeds.

Genomic DNA was purified using the Wizard gDNA extraction kit (Promega, Madison, WI, USA) and sequenced on the GS Junior using Titanium chemistry (Roche Diagnostics, Laval, QC, Canada). Two shotgun sequencing runs were performed. A total of 290,085 reads (134.79 Mbp) were assembled using Newbler v. 2.7 (454 Life Sciences) into 51 large contigs with an *N*_50_ contig size of 324 kbp. Additionally, a paired-end sequencing run was performed based on the paired-end rapid library preparation protocol (8 kb to 20 kb span) for Titanium chemistry (Roche, March 2012), with modifications as described (4). A total of 112,190 paired-end reads was generated with an estimated pair distance of 7,373 ± 1,843 bp. Assembly of all sequencing runs together produced a final high-quality draft sequence (5) featuring 33× genome coverage in 43 large contigs and 4 scaffolds, the largest of which was 6.1 Mbp. Sequence data were annotated using the Prokaryotic Genome Annotation Pipeline v. 2.0 (NCBI).

The genome of *Pseudomonas* sp. BRG100 contained 6,246,661 bp and had a GC content of 59.23%. A total of 5,743 genes and 5,507 protein-coding genes were observed, along with 3 genes encoding 5S rRNA, 1 gene encoding 16S rRNA, 1 gene encoding 23S rRNA, and 57 genes encoding tRNA. The majority (79.77%) of protein-coding genes had function prediction, and 2,021 clusters of orthologous groups (COG) were identified.

Comparison of the genome sequence of *Pseudomonas* sp. BRG100 to 20 other sequenced genomes from various *Pseudomonas* sp. using JSpecies (6) revealed that none of the genomes we included in the analysis had metrics that were above the specified cutoff for inclusion in the same species. The closest match was *P. fluorescens* A506, which had an average nucleotide identity (ANI) based on BLAST (ANIb) of 89.71%, an ANI based on MUMer (ANIm) of 90.50%, and a tetra score of 0.99651. Similarly, SpecI (7) was unable to assign BRG100 to a species cluster (average nucleotide identity was 95.3% to *Pseudomonas fluorescens*). These findings suggest that BRG100 might be a novel species of *Pseudomonas*, or that whole genomes are lacking for the species to which this strain belongs. At the genome sequence level, *Pseudomonas* sp. BRG100 appears to be distinct from *P. fluorescens*, although this appears to be its closest relative among *Pseudomonas* species with a genome sequence in public databases.

### Nucleotide sequence accession numbers.

This whole-genome shotgun project has been deposited at DDBJ/EMBL/GenBank under the accession number JPRX00000000. The version described in this paper is version JPRX01000000.
